# Succession of the Bacterial Communities and Functional Characteristics in Sheep Manure Composting

**DOI:** 10.3390/biology11081181

**Published:** 2022-08-05

**Authors:** Xu Zhao, Juan Li, Zongxian Che, Lingui Xue

**Affiliations:** 1 School of Chemistry and Chemical Engineering, Lanzhou Jiaotong University, Lanzhou 730070, China; 2 Institute of Soil, Fertilizer and Water-Saving Agriculture, Gansu Academy of Agricultural Sciences, Lanzhou 730070, China

**Keywords:** composting, sheep manure, bacterial community, functions

## Abstract

**Simple Summary:**

The conversion of livestock manure into organic fertilizer through composting is an effective way to harmlessly and resourcefully utilize manure. The bacterial communities change rapidly during the composting process, and there is a synergistic effect of various bacteria, which realizes the dynamic fermentation of composting. However, the succession of bacterial communities and its relationship with the physicochemical properties of the material during sheep manure composting remain unclear. In this study, high-throughput sequencing technology and bioinformatics tools were used to analyze the succession and to explore the metabolic functions of bacterial communities during sheep manure composting. The results will help to further improve the sheep manure composting process, improve the quality of compost products, and promote fertilizer utilization of sheep manure.

**Abstract:**

Bacterial community is a key factor affecting aerobic composting, and understanding bacterial community succession is important to revealing the mechanism of organic matter degradation. In this study, the succession and metabolic characteristics of bacterial communities were explored in 45 days composting of sheep manure and wheat straw by using high-throughput sequencing technology and bioinformatics tools, respectively. Results showed that the alpha diversity of bacterial community significantly decreased in the thermophilic (T2) phase and then recovered gradually in the bio-oxidative (T3) and the maturation (T4) phases. Bacterial communities varied at different stages, but there were 158 genera in common bacterial species. *Unclassified_f_Bacillaceae*, *Oceanobacillus*, *Bacillus*, *Pseudogracilibacillus*, and *Nocardiopsis* were identified as keystone bacterial genera. Eleven genera were significantly correlated (*p* < 0.05), or even extremely significantly correlated (*p* < 0.001), with the physicochemical factors. Redundancy analysis (RDA) showed that changes of bacterial community diversity correlated with physicochemical factors. The highest relative abundances were amino acid and carbohydrate metabolism among the metabolic groups in the compost. These results will provide theoretical support for further optimizing sheep manure composting conditions and improving the quality of organic fertilizers.

## 1. Introduction

The consumption of meat increased the intensive development of sheep feeding in China; thus, the amount of sheep manure has risen and needs to be recycled by using different technologies [1]. Sheep manure must be handled with great care, and adverse effects on greenhouse gas emissions (CH_4_, N_2_O) as well as on surface and groundwater contamination must be minimized [2]. Composting is an environment-friendly and useful technology that can effectively reduce the pollution of livestock and poultry manure and convert it into organic fertilizer that can improve the organic matter of arable land. The composting of livestock and poultry manure plays a notable role in maintaining the balance of agroecosystem and sustainable development of agricultural waste [3,4,5]. The composting process degrades and converts organic matter into humus through the physiological metabolic function of microorganisms to produce carbon dioxide, water, nitrate, ammonia, and other low molecular weight compounds [6]. The high temperature generated by composting can effectively kill pathogenic microorganisms and weed seeds and further promote the degradation of organic matter [7].

A large number of microorganisms in composting participate in the harmlessness and recycling of the organic matter [8]. Microbial activity directly affects the decomposing process of compost, and the reproduction and metabolic intensity of microorganisms are important factors for the smooth progress of composting [9]. The decomposing process and composition of the microbial community in composting are affected by environmental factors such as temperature, C/N ratio, pH value, and moisture [10]. Mao et al. [11] found that the dominant flora including *Firmicutes, Proteobacteria, Actinobacteria*, and *Acteroidetes* mainly existed in pig manure composting, but the relative abundances were distinct in different composting stages. Neher et al. [12] found that composting microorganisms with different materials, methods, and composting stages showed different dominant species in classification. Therefore, a better understanding of the microbial community in the compost is critical for improving the quality of the compost product and reducing nutrient losses [13].

During composting, bacteria are more proactive because of the facultative nature of metabolism and their adaptation at an adverse environmental condition. Researchers have revealed that bacterial communities are highly capable of decomposing and mineralizing organic matter in high temperature environments, so they play a very important role in the thermophilic phase of composting [14,15,16]. In addition, bacteria play an important role in the degradation of proteins, lipids, cellulose, lignin, and other organic matter [17,18]. Therefore, the investigation of relative abundance and bacterial dynamics during composting is of great importance to understand the overall mechanism of organic waste mineralization [19]. Although the succession of bacterial communities has been extensively studied in various compostings, due to the different physical and chemical properties of different composting materials, the bacterial species, community structure, and metabolic functions of different composting periods are also different. It is of important practical and theoretical significance to study the relationship between the dynamic changes of bacterial communities and the degradation of organic matter during composting for accelerating the composting process [20]. 

High-throughput sequencing can effectively detect changes of microbial communities, and it has been widely used to study complex microbial communities during the composting process [21,22]. It was highly accurate in using PICRUSt (Phylogenetic Investigation of Communities by Reconstruction of Unobserved States) to predict the metabolic function of the microbial community during composting [23]. Elucidating microbial metabolism and its biochemical characteristics is conducive to deciphering the working mechanism of microbials, which drives efficient transformation and rapid stabilization in the compost [24]. However, there are few reports on the mechanism of sheep manure compost decomposing from the correlation between bacterial community changes, physicochemical factors, and metabolic functions. This study in which composting was constructed using sheep manure and wheat straw under natural conditions, the aim was to explore the ecological functions of bacterial communities during composting and to demonstrate that succession of bacterial communities is associated with changes of physicochemical factors.

## 2. Materials and Methods

### 2.1. Aerobic Composting and Sampling

Three natural composting piles containing sheep manure and wheat straw were prepared at a ratio of 8:1 in Wuwei, Northwest China in May, 2021. Composting piles were approximately 15 m × 2.5 m × 1.5 m (length × width × height). The composting piles had approximately 68% moisture content and a 24:1 C/N ratio. The composting was turned mechanically every 3 days during the first 25 days and then every 5 days until day 45 of the composting. The samples were collected from 6 different points at 2 depths (25 cm, 50 cm from the surface) of the composting piles on days 0, 5, 25, and 45, then samples were mixed and divided into 3 replicates. A portion of the sample was stored at −80 ℃ for the bacterial DNA extraction, the second part was stored at −4 °C for measurement of the ammonium, nitrate, moisture content, seed germination indexes, and pH. The remaining samples were air-dried for measurement of the total nitrogen (TN) and the total organic carbon (TOC). The samples collected on days 0, 5, 25, and 45 were then selected to represent the initial phase (T1), thermophilic phase (T2), bio-oxidative phase (T3), and maturation phase (T4).

### 2.2. Physicochemical Parameters Analysis

The temperature of the center of composting was daily monitored at two times: 8:00 a.m. and 2:00 p.m., respectively. The pH was determined after shaking the fresh samples in water at a ratio of 1:10 (*w/v*) at 180 r/min for 30 min. The moisture content was determined by oven drying to a constant weight at 105 °C [25]. The NH_4_^+^ concentration, NO_3_^−^ concentration, TN, TOC, C/N ratio, and germination index (GI) were determined using the methods described by Sun et al. and Duan et al. [26,27]. 

### 2.3. DNA Extraction and 16S rRNA Gene Sequencing

The extraction of total genomic DNA of compost samples (0.3 g each) were carried out by using the Power Soil DNA extraction kit (MoBio Laboratories Inc., Carslab, CA, USA). All PCR reactions were carried out in 20 μL, with 0.8 μL forward and reverse primers (5 μM), 10 ng template, 4 μL buffer (5×), 2 μL dNTPs (2.5 mM), 0.4 μL polymerase, and 0.2 μL BSA. The PCR products were tested for the concentration, purity, and integrity of DNA, using 1% agarose gel and NanoDrop2000 spectrophotometer (Thermo Fisher Scientific, Waltham, MA, USA). Subsequently, the primer 338 F (5′-ACTCCTACGGGAGGCAGCAG-3′)/806R (5′-GGAC-TACHVGGGTWTCTAAT-3′) was used to amplify the bacterial 16S rRNA, and the Illumina MiSeq PE300 platform was used for high-throughput sequencing [28]. All PCR amplification, library preparation and detection, and computer sequencing analysis were completed by Shanghai Majorbio Biomedical Technology Co., Ltd. Both OTU analysis and sequencing data analysis were performed on the bioinformatics cloud platform (http://www.cloud.majorbio.com/) on 17 November 2021 [15]. The 16S rRNA gene sequence of composting microorganism was submitted to the NCBI database and received the accession number: PRJNA854775.

### 2.4. Bioinformatics and Data Statistical Analysis

Principal component analysis (PCA) was utilized to assess the differences in bacteria communities and their changes during the composting process. Redundancy analysis (RDA) was conducted to reveal the relationship of multiple variations between environmental factors and community composition using the in vegan package of R. Heat maps were drawn using the R heat map package (version 3.3.1). The PICRUSt1 tool and genome (KEGG) database was used to predict bacterial metabolic functions [29]. All bioinformatics analyses were performed using the Majorbio I-Sanger cloud platform. All physicochemical properties and diversity indexes used for analyses were performed in triplicates. All data were analyzed by SPSS version 26.0. The significance level of differences was set at *p* < 0.05. Origin software (v. 2018) was used for data statistics and graphing.

## 3. Results and Discussion

### 3.1. Changes of Physicochemical Properties during Composting

The evolution of physicochemical properties during the composting process of sheep manure are shown in Table 1. Temperature is an important indicator for understanding the composting process. During the initial and thermophilic stages, proteins, amino acids and unstable carbohydrates are rapidly decomposed by microorganisms, generating a large amount of heat in the compost, which speedily increases the temperature of the compost. [30,31]. Composting entered the thermophilic phase (>50 °C) on the 5th day, the maximum temperature was 65.47 °C, and the time that the temperature remained above 50 °C reached 30 days, meeting the requirement that the temperature of the composting be greater than 50 °C and lasting for 7 days [32]. The compost temperature dropped to 36.3 °C on the 45th day. The temperature changes as the energy is released by the microorganisms breaking down organic matter. By detecting the temperature changes in composting, the activity and decomposition degree of the microorganisms can be judged [33]. According to temperature, the composting process is divided into four stages: initial (days 0), thermophilic (days 1–5), bio-oxidative (days 6–25), and maturation (days 26–45).

During the thermophilic phase of composting, microorganisms use soluble organic matter to rapidly reproduce and speedily mineralize organic matter, resulting in a decrease in TOC. [34]. Mesophilic microorganisms decompose macromolecular organics, such as cellulose, hemicellulose and lignin, in composting to reduce TOC. It was found that TOC decreased from 44.45% to 38% with the degradation rate of 13.23%. Whilst, the organic nitrogen in composting was decomposed, the ammoniation was enhanced, the nitrification was inhibited, and thus a large amount of ammonia gas was generated and volatilized, resulting in a decrease in the content of TN and NO_3_^–^-N and an increase in NH_4_^+^-N. In the bio-oxidative phase (T3), the ammoniation gradually weakened, the mineralization increased, TN and NO_3_^−^-N continued to accumulate and began to increase, and the NH_4_^+^-N content was the highest, reaching 2.48 mg·kg^−1^ TS. In the maturation phase (T4), the ammoniation was weakened, the nitrification was enhanced, the contents of TN and NO_3_^−^-N further increased: the TN content increased to 1.91% TS, the NO_3_^−^-N content increased to 0.46 mg·kg^−1^ TS, and the NH_4_^+^-N content was reduced to 0.27 mg·kg^–1^ TS.

Microorganisms absorb nutrients dissolved in water for reproduction and metabolism, and they are spread with water as a carrier to promote the fermentation and degradation of substances [35]. The decrease rate of moisture content (MC) was largest in the T2 and the T3 phases, and it was slower in the T4 phase. The moisture content decreased to 39.78% at the end of composting. 

The effects of pH on the microorganisms led to a change in the charge of the cell membrane, thereby affecting the absorption of nutrients [36]. The pH changes in composting first showed an increasing and then a decreasing trend. Since the decomposition of organic matter by microorganisms produced a large amount of NH_4_^+^, the pH rose rapidly in the stages of T2 and T3. In the T4 phase, the metabolic activity of microorganisms was decreased, the degradation rate of organic matter slowed down, the ammoniation weakened, and the pH decreased and tended to be stable. The pH of each treatment showed a preliminary upward trend, which was caused by the biodegradation of acidic substances and the rapid mineralization and accumulation of nitrogen-containing organic compounds (proteins and amino acids), such as ammonium nitrogen (NH_4_^+^-N) and NH_3_. After the T2 phase, the pH decreased and finally stabilized, which may be due to the emission of ammonia and the generation of organic acids during the decomposition of organic matter in the T3 and the T4 phases.

The C/N ratio is an index to measure the nutritional balance of compost, and it is also an important index to evaluate the maturity of compost [37]. During the composting process, the C/N ratio increased first and then decreased. In the T2 phase, the metabolic activity of microorganisms was enhanced, the organic matter was consumed faster, and a large amount of NH_4_^+^ and NH_3_ were produced, so the TOC and TN content rapidly decrease. The degradation rate of TN was higher than that of TOC and the C/N rise. In the T4 phase, the degradation rate of TOC slowed down, the accumulation of TN increased, and C/N ratio further decreased. It is generally believed that the state of decomposing is reached when C/N ≤ 20 [38] and the C/N of the composting material at the end of composting is 19.8, indicating that the composting material has decomposed.

In the T1 and T2 phases, high concentrations of NH_3_ and organic acids have obvious limiting effects on seed germination. Therefore, the seed germination index (GI) in the T1 and the T2 phases are 22.94% and 15.97%, respectively, which are lower than those of the other phases. With the decomposition of organic acids and NH_3_ volatilization or oxidation to NO_3_^−^, the inhibitory effect was weakened, and microorganisms produced some hormones in the T4 phase that promote plant growth, such as auxin, gibberellin, etc. [39]. The GI was gradually increased and reached 76.6% at the end of composting. Generally speaking, when GI > 70%, it can be considered that the compost is basically non-toxic to plants, indicating that the compost has been decomposed [40].

### 3.2. Alpha Diversity of Microbial Community during the Composting Process 

Chao1 and ACE indexes are used to evaluate the abundance of microbial communities, and Shannon and Simpson indexes are used to evaluate the diversity of microbial communities. The larger index indicated the higher abundance or diversity of microbial communities [41]. Table 2 shows the alpha diversity index of the bacterial community in different phases. The coverage index was greater than 99%, indicating that the gene sequence has a high probability of being detected, the sampling was reasonable, and it could truly reflect the bacterial communities of the compost samples. The ACE and Chao1 indexes of the bacterial communities at different stages were significantly different, indicating that there were significant differences in the abundance of the bacterial communities in different phases. The ACE and Chao1 indexes were the highest in the T3 phase and the lowest in the T4 phase. The Simpson and Shannon indexes were different in various compost phases, indicating that the diversity of the bacterial communities was different in various phases. The Simpson index was the highest in the T3 phase and the lowest in the T2 phase; in the T2 and the T3 phases, the Simpson was significantly different from other phases. The Shannon index was the highest in the T2 phase and the lowest in the T3 phase. In the T1 and the T2 phases, the Shannon was significantly different from other phases.

**Figure 1:**
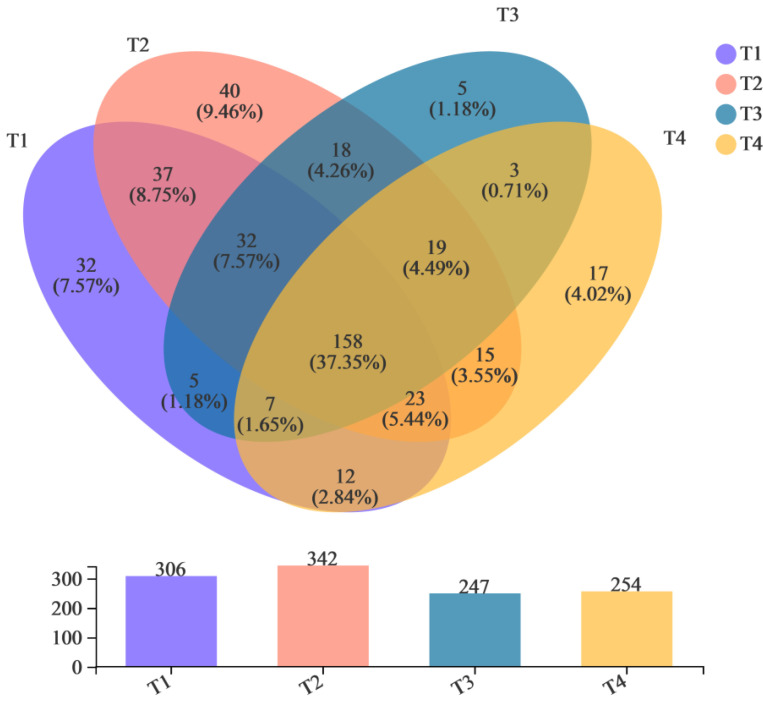
Venn diagram of bacterial communities.

The sequences of samples at different stages of composting were clustered into Operational Taxonomic Units (OTUs), according to 97% consistency, and then analyzed. Figure 1 shows the Venn diagram of bacterial communities of samples in various stages of composting. Different colors represent different groups. The numbers in the overlapping parts indicate the number of species in common in the groups, and the numbers in the non-overlapping parts indicate the number of species unique to a group. There were 306 genera of microorganisms in the T1 phase and 342, 247, and 254 genera in the T2, T3, and T4 phases, respectively. There were 158 genera of common microorganisms in various stages. Similarly, many scholars have found that different core microbial groups exist in the composting process of different raw materials [42,43,44]. Above all, this suggests that although the alpha diversity of microorganisms was highest in the initial stage, most species were not involved in the compost degradation process. During the composting process, microbial groups that do not participate in composting fermentation will be eliminated, and functional microbial groups suitable for composting fermentation of different raw materials will be formed.

### 3.3. Bacterial Community Succession during the Composting Process

**Figure 2:**
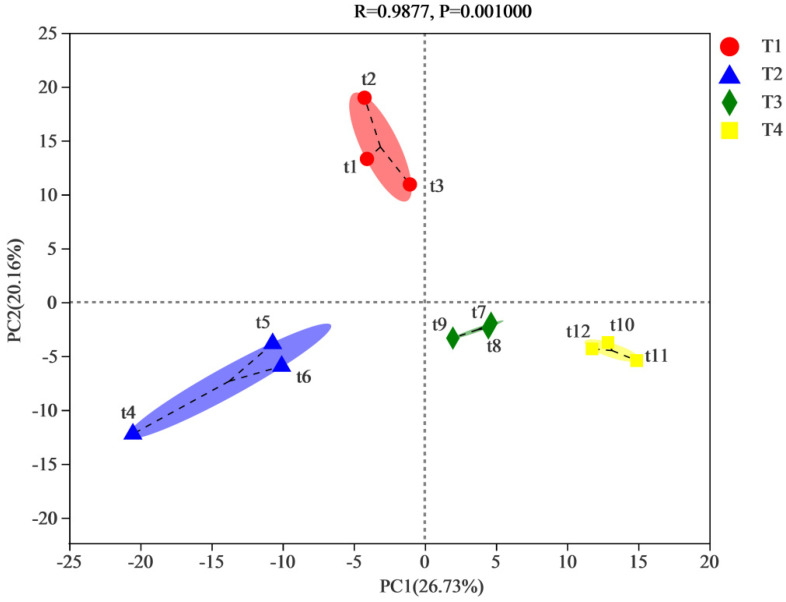
Principal component analysis (PCA).

The whole profile and the critical dispersion of bacterial separation for the different phase of the compost process was investigated via adopting PCA, as illustrated in Figure 2. It was calculated that the bacterial genus number among all phases and the closer distance indicated the more similar bacterial community composition. The principal component (PC1) explained 26.73% of the total bacterial variability in the four phases for X-axis. The results implied that the bacterial communities of T3 and T4 were similar and that T1, T2 and T3, T4 were divided into different bunches. These observed results also illustrated that the phase had a great impact on bacterial diversity. Meanwhile, there was a significant effect on the bacterial community between a higher temperature (T2) and a lower temperature (T1, T3, T4). Obviously, different phases had directly changed environmental parameters, resulting in differences in bacterial diversity structure.

**Figure 3:**
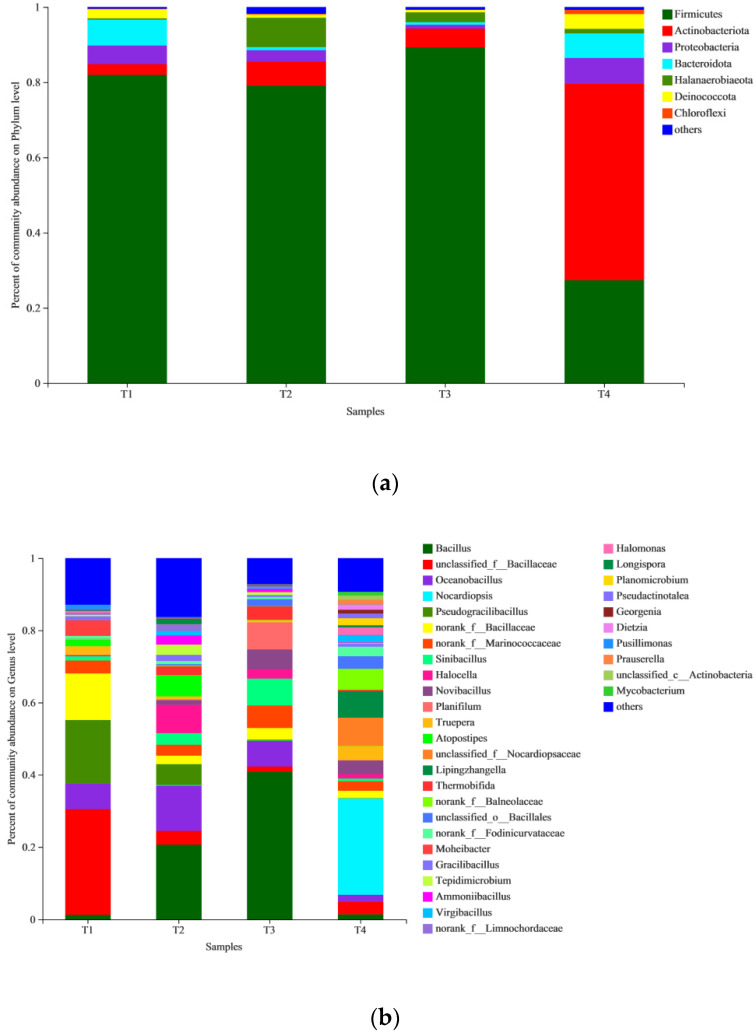
(**a**) A diagram of relative bacterial abundance at phylum level. Phyla with relative abundance < 1% were combined and indicated as “others”. (**b**) A diagram of relative bacterial abundance at genus level. Genera with relative abundance < 1% were combined and indicated as “others”.

At the phylum level, seven bacterial phyla were observed in the different phases of the composting process (Figure 3a). *Firmicutes* was the dominant phylum in the T1, T2, and T3 phases. *Firmicutes* are fermenting bacteria that can form heat-resistant spores in high temperature environments, and they play an important role in decomposing organic matter in the thermophilic stage of composting [45]. *Actinobacteriota* was the dominant phylum in the T4 phase. Because *Actinomycetes* produce carbohydrate-active enzymes that break down lignocellulose, they play an important role in the process of breaking down lignin in the later stages of composting [46]. In initial phase (T1), there were 5 phyla with relative abundance > 1.0%, and 2 phyla with relative abundance > 5.0%, that was, *Firmicutes* (81.94%), *Bacteroidota* (6.90%), *Proteobacteria* (4.94%), *Actinobacteriota* (2.86%), and *Deinococcota* (2.43%). These predominant bacterial phyla were identified in many kinds of organic waste composting [19,47,48]. There were 4 phyla with relative abundance > 1.0%, and 3 phyla with relative abundance > 5.0%, namely *Firmicutes* (79.09%), *Halanaerobiaeota* (7.82%), *Actinobacteriota* (6.36%), and *Proteobacteria* (3.06%) in the thermophilic phase (T2). There were 4 phyla with relative abundance > 1.0%, and 2 phyla with relative abundance > 5.0%, namely *Firmicutes* (89.24%), *Actinobacteriota* (5.00%), *Halanaerobiaeota* (2.63%), and *Proteobacteria* (1.01%) in the bio-oxidative phase (T3). There were 7 phyla with relative abundance >1.0% and 4 phyla with relative abundance >5.0%, namely *Actinobacteriota* (52.22%), *Firmicutes* (27.32%), *Proteobacteria* (6.88%), *Bacteroidota* (6.55%), *Deinococcota* (3.92%), *Halanaerobiaeota* (1.21%), and *Chloroflexi* (1.15%) in the maturation phase (T4). The Firmicutes phylum significantly decreased in the maturation phase (T4) but *Actinobacteriota* and *Deinococcota* increased significantly. These are similar to the findings of Wei et al.; since the relative abundance of *Actinomycetes* in the maturation phase was higher, it is inferred that *Actinomycetes* is an important indicator for judging the maturity of composting [49].

The microbial abundance at the genus level can reveal the succession of dominant microorganisms during the composting process [12]. The changes in the composition of bacterial genus-level communities in different phases of composting are shown in Figure 3b. The dominant taxa (relative abundance >1.0% in samples from at least one composting period) were: *unclassified_f__Bacillaceae, Pseudogracilibacillus, norank_f__Bacillaceae, Oceanobacillus, norank_f__Marinococcaceae, Atopostipes, Bacillus, Sinibacillus, Gracilibacillus, Moheibacter, Truepera, Pusillimonas, Halocella, Tepidimicrobium, Ammoniibacillus, Thermobifida, Virgibacillus, norank_f__Limnochordaceae, Longispora, Novibacillus, unclassified_o__Bacillales, Planifilum, Nocardiopsis, unclassified_f__Nocardiopsaceae, Lipingzhangella, norank_f__Fodinicurvataceae, norank_f__Bacillaceae, Halomonas, Planomicrobium, Virgibacillus, Dietzia, Prauserella, Pseudactinotalea, unclassified_c__Actinobacteria, Georgenia, Mycobacterium,* and 35 other genera. Bacterial community structure varies in different phases of composting. In the initial phase (T1), there were 12 genera with relative abundance > 1.0%, and 4 genera with relative abundance > 5.0%, namely *unclassified_f__Bacillaceae* (29.19%), *Pseudogracilibacillus* (17.56%), *norank_f__Bacillaceae* (12.89%), and *Oceanobacillus* (7.09%). In the thermophilic phase (T2), there were 17 genera with relative abundance > 1.0%, and 5 genera with relative abundance > 5.0%, namely *Bacillus* (20.62%), *Oceanobacillus* (12.52%), *Halocella* (7.82%), *Atopostipes* (5.90%), and *Pseudogracilibacillus* (5.77%). In the bio-oxidative phase (T3), there were 11 genera with relative abundance > 1.0%, and 6 genera with relative abundance > 5.0%, namely *Bacillus* (40.79%), *Planifilum* (7.50%), *Sinibacillus* (7.45%), *Oceanobacillus* (7.02%), *norank_f__Marinococcaceae* (6.22%), and *Novibacillus* (5.46%). In the maturation phase (T4), there were 23 genera with relative abundance > 1.0%, and 4 genera with relative abundance > 5.0%, namely *Nocardiopsis* (26.65%), *unclassified_f__Nocardiopsaceae* (7.75%), *Lipingzhangella* (7.36%), and *norank_f__Balneolaceae* (5.83%). Changes in temperature affect the metabolic activities of microorganisms and the succession of community structure [50]. With the increase of temperature, the bacterial taxon increased. However, when the temperature rose continuously, the bacterial taxon gradually decreased and reached the minimum in the thermophilic phase. After the thermophilic phase, with the decrease of compost temperature, the bacterial taxon began to increase again.

### 3.4. Correlation between Relative Abundance of Bacterial Genera and Physicochemical Factors

**Figure 4:**
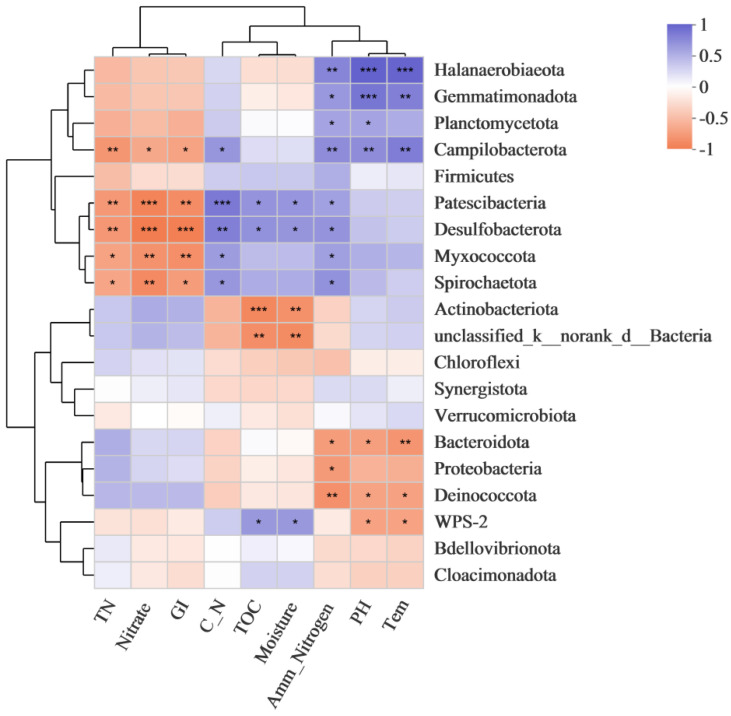
Spearman correlation between dominant genera and physicochemical factors during compost process. The correlation coefficient is represented by the color and size of the circles. Dark red indicates positive correlation and dark blue indicates negative correlation. *p* values were calculated using Spearman’s rank correlation test, * *p* < 0.05; ** *p* < 0.01; *** *p* < 0.001.

The composition and distribution of microbial communities are diverse due to the influence of environmental factors, so there is a certain correlation between the diversity of microbial communities and environmental factors [51]. The Spearman correlation coefficient was used to evaluate the correlation between the top 20 species in total abundance of bacterial genus and the physicochemical factors. Figure 4 exhibits eleven genera, including *Halanaerobiaeota, Gemmatimonadota, Campilobacterota, Patescibacteria, Desulfobacterota, Myxococcota, Spirochaetota, Actinobacteriota, unclassified_k_norank_d_Bacteria, Bacteroidota,* and *Deinococcota* were significantly correlated (*p* < 0.05), or even extremely significantly correlated (*p* < 0.001), with the physicochemical factors. *Halanaerobiaeota, Gemmatimonadota*, and *Campilobacterota* were positively correlated with temperature, pH, and Amm_Nitrogen. *Halanaerobiaeota* was very significantly positively (*p* < 0.001) correlated with pH and temperature and significantly positively (*p* < 0.05) correlated with Amm_Nitrogen. *Gemmatimonadota* was very significantly positively (*p* < 0.001) correlated with pH, significantly positively (*p* < 0.05) correlated with temperature. *Campilobacterota* was significantly positively (*p* < 0.05) correlated with pH, temperature, and Amm_Nitrogen. *Patescibacteria, Desulfobacterota, Myxococcota,* and *Spirochaetota* were negative correlated with TN, GI, and nitrate. *Patescibacteria* was very significantly positively (*p* < 0.001) correlated with C/N, very significantly negatively (*p* < 0.001) correlated with nitrate, and significantly negatively (*p* < 0.05) correlated with TN and GI. *Desulfobacterota* was significantly positively (*p* < 0.001) correlated with C/N, and very significantly negatively (*p* < 0.001) correlated with nitrate and GI. *Myxococcota* was significantly negatively (*p* < 0.05) correlated with nitrate and GI. *Spirochaetota* was significantly negatively (*p* < 0.05) correlated with nitrate.

**Figure 5:**
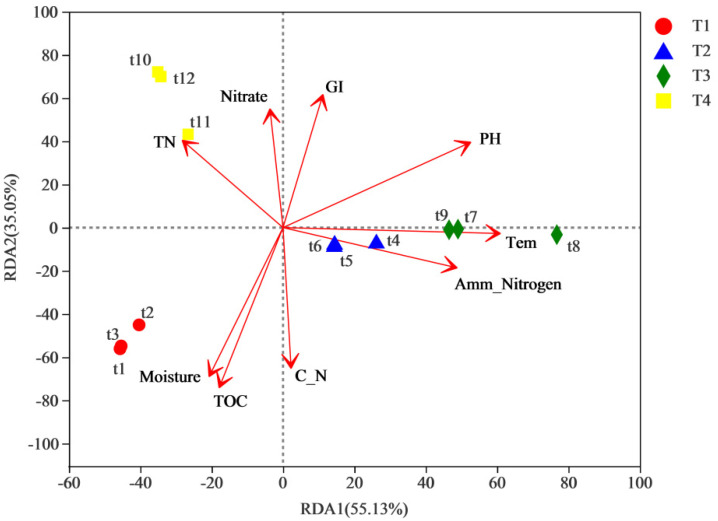
Multivariate redundancy analysis of bacterial and chemical properties (arrows) at different phases of composting.

Redundancy analysis (RDA) was performed to further analyze the relationships between physicochemical properties and the bacterial communities during different composting phases. The bacteria in the different phases were divided into four clusters (Figure 5). In the composting system, the selected explanatory variables accounted for 90.18% of the redundancy, where the “axis 1” explained variation and the “axis 2” explained variation were 55.13% and 35.05%, respectively. The correlation between physicochemical properties and bacterial communities was illustrated as follows: Tem > pH > TOC≈Amm-Nitrogen > moisture > TN > C_N≈GI > TN > nitrate. Based on the bacterial community, moisture and TOC were the key factors in the T1 phase; temperature and amm-nitrogen became the main influencing factors for the T2 and the T3 phases; and TN and nitrate became the main influencing factors for the T4 phase. Generally, temperature was the most important environmental factor during composting [52,53]. Therefore, the physicochemical properties could significantly affect the succession of bacterial communities during composting. RDA analysis showed that the bacterial community was clearly influenced by chemical parameters during the compost process, indicating that the former community was more sensitive to environmental fluctuations.

### 3.5. Bacterial Function Predictions

**Figure 6:**
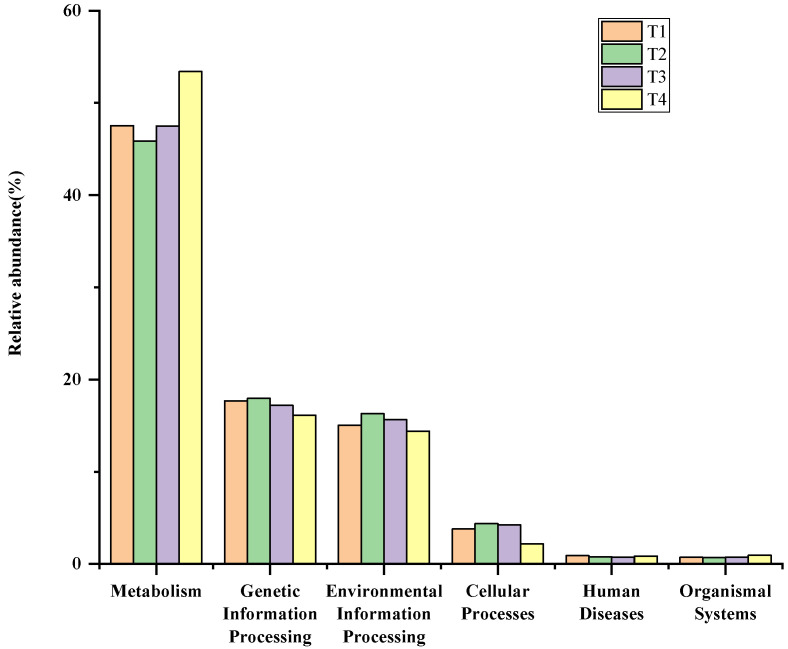

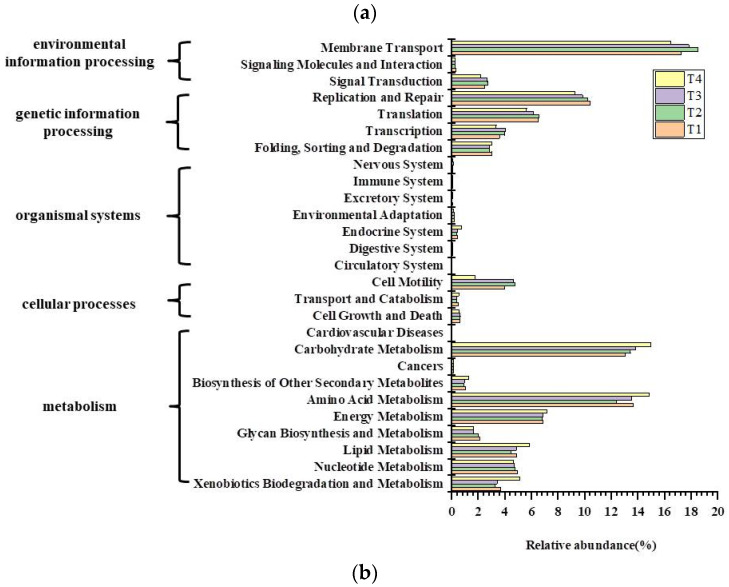
(**a**) KEGG metabolic pathway level 1 characteristics of bacteria. (**b**) KEGG metabolic pathway level 2 characteristics of bacteria.

The bacterial functions of composting microorganisms were predicted by PICRUSt2 based on the KEGG pathway, as shown in Figure 6a,b. Most of the predicted pathways based on the relative abundant bacterial sequences in compost samples could be divided into six functional groups (pathway level 1): metabolism (47.52–53.4%), environmental information processing (14.41–16.28%), genetic information processing (16.14–17.95%), cellular processes (2.18–4.37%), human diseases (73.61–89.17%), and organismal systems (68.23–93.74%) (Figure 6a). The level 2 KEGG function predictions included 10 pathways for metabolism, 7 for organismal systems, 4 for genetic information processing, 3 for environmental information processing, and 3 for cellular processes (Figure 6b). The main metabolic pathways were carbohydrate metabolism (13.04–14.97%) and amino acid metabolism (12.41–14.87%), which was similar to previous reports of composting [52,54].

The amino acid, carbohydrate, energy, lipid, cofactors, and xenobiotics biodegradation metabolism were highest in the T4 phase of composting. However, the genetic information processing (transcription, replication, and repair) metabolic functions of the T4 phase were lowest. In this phase, the composting temperature was moderate, which was more suitable for the production of metabolites. Yet, the bacterial community was few, so the transmission of genetic information was lower in the T4 phase. The deamination of amino acids leads to the faster degradation of TN in samples during the T2 phase, which was more conducive to the nitrogen and phosphorus cycle of plants. The samples in the T1, T2, and T3 phases had certain advantages in environmental information processing (membrane transport), and the gene abundance was increased compared with samples of the T4 phase, indicating that composting helps to promote membrane transport and exchange of beneficial factors in and out of microorganisms, resulting in the growth of microorganisms.

The highest relative abundances were amino acid and carbohydrate metabolism in the maturation phase among the metabolic groups. These results showed that the bacterial metabolic group of amino acids can increase the amount of amino acids and humic acids [55]. The synthesis of humus and the production of amino acids during composting are closely related to the amino acid metabolic intensity of bacteria [56]. The proportions of carbohydrate and amino acid metabolism occupied >10% in the entire composting process. The relative abundance of carbohydrate and lipid metabolism dramatically increased after the bio-oxidative phase of composting, amino acids, and energy metabolism slightly decreased in phase T2 and later increased again, coinciding with the finding of Wei’s group [50]. Carbohydrate metabolism played vital roles in lignocellulosic, hemicellulose, and cellulose degradation. Lignocellulosic, hemicellulose, and cellulose in compost biodegradation produce different substances through carbohydrate metabolic pathways [57].

## 4. Conclusions

The changes in bacterial communities and functions during sheep manure composting were investigated in detail. The bacterial community structure and key groups were differed in different stages, and bacterial groups were related to the physicochemical properties of compost materials. The changes of functions are related to bacterial community succession. Carbohydrate and amino acid metabolism were the main metabolic pathways in the composting. Our studies will provide theoretical information for better understanding of material changes during sheep manure composting and optimization of composting conditions.

## Figures and Tables

**Table 1:** Physicochemical properties of the compost in different phases.

Samples	Temperature (℃)	Moisture (%)	TOC (% TS)	TN (% TS)	NO_3_^−^N (mg kg^−1^ TS)	NH_4_^+^-N (mg·kg^−1^ TS)	C/N	pH	GI (%)
T1	32.67 ± 1.46 d	68.02 ± 1.62 a	44.45 ± 0.66 a	1.83 ± 0.036 bc	0.28 ± 0.005 c	0.52 ± 0.102 c	24.3 ± 0.83 ab	9.15 ± 0.06 d	22.94 ± 2.1 c
T2	65.47 ± 1.2 a	62.53 ± 2.11 b	42.46 ± 0.59 b	1.69 ± 0.015 d	0.18 ± 0.009 d	2.48 ± 0.103 a	25.17 ± 0.5 a	9.52 ± 0.06 a	15.07 ± 0.71 d
T3	52.67 ± 1.39 b	45.36 ± 0.75 c	39.75 ± 0.63 c	1.84 ± 0.03 b	0.39 ± 0.015 b	1.29 ± 0.151 b	21.61 ± 0.3 c	9.44 ± 0.02 b	65.97 ± 2.52 b
T4	36.3 ± 0.78 c	39.78 ± 1.88 d	38.00 ± 0.7 d	1.92 ± 0.036 a	0.46 ± 0.016 a	0.27 ± 0.054 d	19.8 ± 0.66 d	9.39 ± 0.01 bc	76.6 ± 2.08 a

Note: Different lowercase letters in each column indicate significant differences in physicochemical parameters in different stages of composting (*p* < 0.05).

**Table 2:** Bacterial richness and diversity in composting samples.

Samples	Simpson	Shannon	ACE	Chao1	Coverage/%
T1	0.060 ± 0.012 bc	3.889 ± 0.151 b	722.143 ± 96.433 ab	696.677 ± 36.616 ab	99.19 ± 0.05 c
T2	0.033 ± 0.002 d	4.311 ± 0.125 a	774.075 ± 31.249 a	767.328 ± 41.332 a	99.13 ± 0.02 cd
T3	0.109 ± 0.011 a	3.284 ± 0.125 cd	680.684 ± 74.245 bc	570.847 ± 65.211 c	99.3 ± 0.07 bc
T4	0.094 ± 0.040 ab	3.545 ± 0.324 bc	608.342 ± 112.575 bcd	544.9 ± 73.681 cd	99.36 ± 0.086 ab

Note: Different lowercase letters in each column indicate significant differences between the same bacterial diversity indices (*p* < 0.05).

## Data Availability

The RNA-seq data is also stored in NCBI with the accession number: PRJNA854775.
